# A Cross-Sectional Survey of the Nutritional Quality of Quinoa Food Products Available in the Italian Market

**DOI:** 10.3390/foods12081562

**Published:** 2023-04-07

**Authors:** Francesca Melini, Valentina Melini, Myriam Galfo

**Affiliations:** CREA Research for Food and Nutrition, Via Ardeatina 546, I-00178 Roma, Italy; francesca.melini@crea.gov.it (F.M.); myriam.galfo@crea.gov.it (M.G.)

**Keywords:** quinoa, gluten-free, nutrition claims, labelling, pseudocereals, vegan, organic, novel food

## Abstract

Quinoa’s (*Chenopodium quinoa* Willd.) status has been recently raised from staple food crop confined to its region of origin to a globally recognized commercial food crop, widely traded in the international market. Claims on food labels may attract consumers who can therefore purchase products with nutrition, allergy/intolerance, or social and ethical claims in an effort to make healthier and more sustainable food choices. The aim of this work was (i) to investigate the nutritional quality of quinoa food products available to the Italian consumer over the e-commerce market, as emerged from nutrition labelling, and (ii) to inquire about the occurrence of nutrition, allergy and intolerance, and social and ethical claims on the packaging. To this aim, a cross-sectional survey of quinoa food products available in the Italian market was conducted. It emerged that several quinoa product categories are available and grains and pasta are the major ones. Nutrition claims are generally displayed in combination with gluten-free and social/ethical claims. Based on the nutrition facts, a higher proportion of products are eligible for nutrition claims. The comparison between the gluten-free labelled and gluten-containing quinoa products showed limited differences in the nutritional quality.

## 1. Introduction

Indigenous to the Andean region (Peru, Bolivia, Ecuador, Argentina, Colombia, and Chile) [1], quinoa (*Chenopodium quinoa* Willd.) has been long considered a minor crop and a neglected and underutilized species with a high potential of development [2]. Its production and consumption had been negligible outside the Andean territory until the late 20th century [3], when global interest was increasingly taken in quinoa’s resilience to agro-ecological extremes (e.g., soil, rainfalls, low temperature, and altitude), tolerance to abiotic stress (e.g., frost, drought, and salinity), and adaptability to environments [2].

After its entry in the European catalogue for novel foods [2,4] and following the FAO International Year of Quinoa in 2013 [5], quinoa’s status was raised from a staple food crop confined to its region of origin to a globally recognized commercial food crop widely traded in the international market [6]. According to estimates, the global area harvested has increased steadily from 47,585 ha in 1990 to 191,676 ha in 2021, with a subsequent increase in quinoa production. In 2021, global quinoa production encompassed 147,037.78 tons, with respect to 23,012 tons in 1990 [7].

Peru, Bolivia, and Ecuador are the main quinoa-producing countries [8]. However, over the past years, cultivation has spread to Northern America, Europe, Asia, and Africa. As for Europe, there were no cultivated areas in Europe until 2008 but quinoa cultivation regions reached 5000 ha in 2015 [9]. Current domestic production, nevertheless, does not manage to meet consumer demand; hence, Europe is the largest global importer of quinoa, accounting for roughly one third of the total import from Andean countries [8]. The total European import in 2019 encompassed 28 thousand tons, 4.5 fold higher than in 2012 [8].

The increased consumption of quinoa and quinoa food products in Europe is linked to consumers’ perception of its quality and safety. Consumers’ food choices are governed by a combination of biological and physiological determinants, as well as by psychological, social, and economic factors [10]. Additional rationales for consumers’ choices/purchase decisions are health benefits, animal welfare, and environmental protection and sustainability [11].

Over the last decades, upon globalization, producers sell packaged goods in large quantities to consumers that live in very distant places; as a consequence, there is no more a personal contact between the food producer and the buyer, and product packaging and labels become the calling card of the product, as well as the tie between the producer and the consumers [12]. Nutrition claims, allergy/intolerance claims, and social/ethic logos enhance consumer knowledge and empower them to make more informed choices.

The aim of this work was (i) to investigate the nutritional quality of quinoa food products available on the online shop websites of the major food retailers present in the Italian market, as emerged from nutrition labelling, and (ii) to inquire about the occurrence of nutrition, allergy and intolerance, and social and ethical claims on the packaging. To the best of our knowledge, no study has so far been carried out to investigate the quinoa domestic market and the nutrition facts of quinoa products. This study specifically surveys the variability of nutrition facts within and among the main categories of quinoa products and investigates the availability of nutrition, allergy/intolerance, and social/ethical claims, as declared on the packaging. A special attention is paid to the differences in the nutritional composition of gluten-free (GF) labelled and gluten-containing quinoa products.

## 2. Materials and Methods

### 2.1. Data Collection

An online survey was conducted from September 2022 to December 2022 on the website of the major food retailers present in the Italian market. The term “quinoa” was used for the search. Products were selected when (i) the term “quinoa” was included as part of the product’s descriptive name, (ii) they were available for purchase in at least one online shop, and (iii) the nutrition facts were available. Products with either no nutrition facts or with unclear/incomplete images of nutrition facts were not collected. For instance, quinoa beer was not included in the dataset due to lack of nutrition facts.

The data were pasted into Microsoft^®^ Excel^®^ for Windows 365 (version 2103), and a dataset was created. Each product was assigned to a product category based on product name and/or composition, i.e., biscuits, bread, bread substitutes, breakfast cereals, flours, grains, pasta/couscous, ready-to-cook products, snacks, and yogurt-like products. Accuracy was double-checked and inaccuracies were resolved through a further data check.

### 2.2. Data Extraction

Data on brand name, descriptive name, and nutrition declaration were recorded for each food item. Energy (kcal/100 g), total fat (g/100 g), saturates (g/100 g), carbohydrate (g/100 g), sugars (g/100 g), protein (g/100 g), fibre (g/100 g), and salt (g/100 g) are mandatory information, as laid down by Council Regulation (EU) No. 1169/2011 [13].

The presence of (i) nutrition claims (NC), as laid by Regulation (EC) No. 1924/2006 [14]; (ii) organic declaration, as laid down by the Regulation (EC) No. 834/2007 [15]; and “gluten-free” and/or “very low gluten” claims, as regulated by the Regulation (EU) No. 828/2014 [16], were also registered. Furthermore, the availability of the “vegan” logo on the packaging was recorded.

The list of ingredients and the percentage of quinoa were also reported in the dataset.

### 2.3. Statistical Analysis

All statistical analyses were performed with Minitab Pro 18 (Minitab Inc., State College, PA, USA). Microsoft^®^ Excel^®^ for Windows 365 (version 2103) was also used to process experimental data.

## 3. Results and Discussion

### 3.1. Categorization of Quinoa Food Products Available on the Italian Online Market

**Figure 1:**
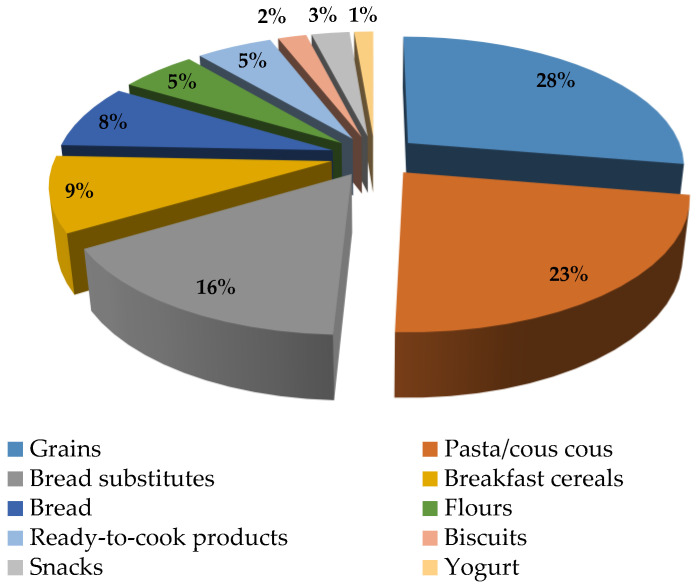
Distribution of quinoa products across product categories.

A total of 150 quinoa food products were identified and grouped into ten product categories (Figure 1). Grains and pasta were the main categories, comprising 28% and 23% of total food items, respectively (Figure 1), followed by bread substitutes (16%), breakfast cereals (9%), and bread (8%). Flours accounted for 5% of the total, as well as ready-to-cook products. Snacks (3%), biscuits (2%), and yogurt (1%) were the least represented product categories.

This shows that quinoa is a versatile food and can be included in the daily diet to contribute to having a varied diet. Quinoa can be, in fact, consumed whole in grain mixtures, in salads, in ready-made meals. It can be flaked, puffed, or extruded to be consumed as breakfast cereals, granola, or energy bars. When it is ground to flour, it is an ingredient for baked products, pastas, and snacks. Technologies are also being optimized to produce quinoa milk- and yogurt-type drinks [17]. Recently, in the framework of planet and diet sustainability, quinoa has been used to formulate vegetable meat alternatives [18]; however, only a small number of products are currently available on the market.

Within each product category, the presence of claims and/or logos was surveyed. Besides nutrition claims, product packaging can also display logos for special diets, such as the gluten-free diet (GFD), and social and ethical diets, with the dietary choices of the latter ranging from organic to vegetarian and vegan [19]. The size of products displaying: (i) no claims; (ii) only nutrition claims (NC); (iii) only allergy or intolerance claims (AIC); (iv) only social and ethical claims (SEC); (v) NC and AIC (NC/AIC); (vi) NC and SEC (NC/SEC); (vii) AIC and SEC (AIC/SEC), or (viii) NC, AIC, and SEC (NC/AIC/SEC) was thus evaluated (Table 1). The NC included all the claims laid down by Regulation (EC) No. 1924/2006 [14], whereas the AIC included the gluten-free claim. Products displaying “organic” and “vegan” logos were classified as reporting social and ethical claims.

The analysis of the presence of claims on the packaging of the collected items showed that 13% of quinoa food products has no claim, whereas the remaining 87% show at least one claim, either nutrition and/or allergy and intolerance, and/or social and ethical, or combination thereof (Table 1). As regards nutrition claims, it emerged that they were shown (i) alone on 4% of total products; (ii) together with AIC on 3% of total products; (iii) together with social/ethical claims on 5% of products, and (iv) together with AIC and SEC on 17% of products (Table 1). Most nutrition claims present on the packaging were “source of fibre”, “high fibre”, “source of protein”. Other claims, such as “low in sodium/salt” and “with no added sugars”, were displayed on the packaging of only a few products.

Claims for special allergy and intolerance purposes (i.e., gluten-free), either alone or in combination with other claims, were present on 59% of products. In detail, the gluten-free logo was present alone in 15% of products, together with NC in 3% of products, together with SEC in 24% of product packaging, and together with the AIC and SEC in 17% of products (Table 1).

As regards the occurrence of social and ethical claims, it was found that 20% of products displayed only “vegan” and/or “organic” logos, whereas a further 46% of products showed SEC in combination with the other claims (NC and AIC) (Table 1).

### 3.2. Nutritional Characteristics and Claims of Quinoa Products

The nutritional composition and the occurrence of nutrition, allergy/intolerance and/or social/ethical claims were investigated and discussed within each product category. Moreover, the matching between nutrition declaration and claims was considered.

#### 3.2.1. Grains

The survey showed that quinoa is mainly available on the Italian market as grains. They can be formulated as dry or canned/steamed grains. Moreover, mixtures of quinoa and other cereals/pseudocereals/legumes are available.

Within “dry grains”, quinoa from white ecotypes is mostly sold. Larger-sized seeds grown on the Bolivian altiplano or highlands, referred to as *Quinoa Real*, are also available. Packages of red or black quinoa are, on the other hand, rarely available; they represent only 8% each of the total number of “dry grains”. However, it was observed that the market offers blends of white and pigmented quinoa seeds, made of white quinoa for ≈50%, red quinoa for ≈30% and black quinoa for ≈20% of the total mixture. From a nutritional point of view, the availability of these mixtures is of interest because scientific studies have shown that pigmented varieties, besides being whole grains and thus contributing to the recommended intake of dietary fibre (DF), are also richer in phenolic compounds, flavonoids, and anthocyanins than white ecotypes [20].

The survey also showed that mixtures of quinoa and other grains are available. Wholegrain and/or pigmented rice, einkorn, oat, barley, and rye are the most common cereals mixed with quinoa. Legumes, especially lentils, are blended with quinoa as well. Quinoa is present in these mixtures in diverse percentages, ranging from 4 to 15%.

On average, the “grains” category presented a median energy content of 362 kcal/100 g, with differences among the three identified sub-categories: in canned/steamed quinoa the median energy content was 79 kcal/100 g, in grain mixtures it was 345 kcal/100 g, and in dry quinoa it was 368 kcal/100 g (Table 2). In a framework of substituting quinoa grains with pasta products so as to have a varied diet, it can be observed that the median energy content of quinoa containing pasta (355 kcal/100 g) was statistically not different (*p* > 0.05) from grains (Table 2).

Considering macronutrients, the content of total fat differed among the grain subcategories (*p* < 0.05), with the highest content in dry quinoa (Table 2). In the three grain subcategories, no significant (*p* < 0.05) differences were observed for total carbohydrates, whereas sugars were lowest in grain mixtures. Dry quinoa seeds are the major source of protein.

As a pseudocereal, quinoa has a well-balanced essential amino acid composition and possesses an excellent protein quality. It is considered as one of the best sources of vegetable proteins as the biological value is similar to that of casein (∼73%) [21]. Moreover, quinoa is rich in lysine and thus complements cereals that are high in sulphur-containing amino acids but low in lysine [22]. The consumption of grain mixtures, such as quinoa, cereals, and legumes, should be encouraged since pseudocereals/legumes and cereals complement each other in terms of protein quality. They also meet the dietary recommendation for a higher DF intake.

The median dietary fibre content was highest in dry quinoa (7.00 mg/100 g) and lowest in canned/steamed grains (2.65 mg/100 g). The comparison between the median DF content of quinoa grains with quinoa pasta products showed the major contribution of the first to daily DF intake. A recent review of the scientific literature shows a deficiency of dietary fibre in people adhering to a gluten-free diet [23]. Hence, the inclusion of quinoa grains in special diets, such as GF ones, might contribute to lowering the risk of dietary fibre deficiencies. Regarding salt content, canned/steamed quinoa is the richest, with a median of 0.75 g/100 g, whereas no statistical differences (*p* < 0.05) were found between dry quinoa and grain mixtures.

The analysis of claims highlighted that SEC and the combination of AIC and SEC are the most displayed ones on the packaging of quinoa grains (Table 1). The “vegan” logo appeared on 6 out of 42 packets of grains in combination with either NC or AIC. The “organic” logo was displayed on the packaging of 25 out of 42 products, of which 11 were alone and 14 were in combination with other claims. As regards the “gluten-free” claim, it emerged that 18 out of 42 quinoa grains were claimed as GF to mark that these grains and blends thereof with GF cereals such as rice or legumes are suitable for coeliacs.

Nutrition claims are, on the other hand, reported on only 5% of the products. The NC “source of fibre”, “high in fibre”, and “source of protein” are the most common; other claims, e.g., “low sodium/salt”, “high phosphorous”, “high magnesium”, and “high vitamin E”, are rarely reported on the packaging.

Overall, the analysis of the claims and the cross-check with the nutrition facts confirm that quinoa grains are a significant source of protein and DF; hence, their inclusion in some diets, such as gluten-free and vegetarian ones, can be of paramount importance to prevent deficiencies.

**Figure 2:**
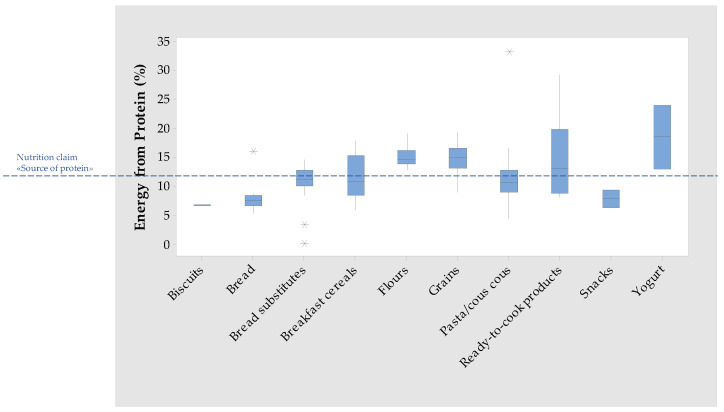
Boxplot for energy from protein (%) in quinoa food categories and reference value for the claim “source of protein”.

For people following a GF diet, the protein supply by quinoa grains is important because GF products have been generally considered low in protein content [23]. Figure 2 shows that the median and the first quartile values of energy from protein (calculated by assuming that each gram of protein contributes 4 kcal) in the collected quinoa grains are higher than 12%, which is the reference value for the claims “source of protein”, as laid down by Regulation (EC) No. 1924/2006 [14]. Hence, most of the quinoa grains are a source of protein and may contribute to increasing protein intake in coeliac subjects. The same trend was observed in flours and yogurts, whereas bakery products, snacks, and pasta showed a median value lower than the reference intake.

**Figure 3:**
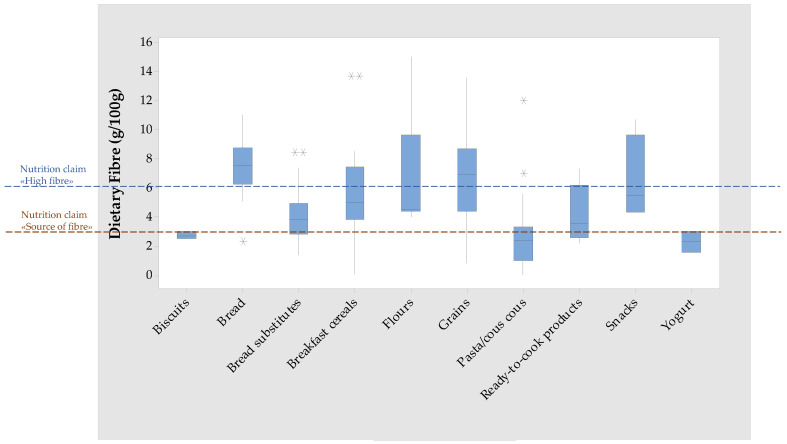
Boxplot for dietary fibre (g/100 g) in quinoa food categories and reference values for the claims “source of fibre” and “high fibre”.

Moreover, the inclusion of quinoa grains can guarantee a high intake of DF. Figure 3 shows the distribution of DF content among quinoa food products and the two reference values for the claims “source of fibre” and “high fibre”, laid down by Regulation (EC) No. 1924/2006 [14]. As regards the grain category, the median value is higher than 6 g/100 g (high fibre) and the first quartile is higher than 3 g/100 g (source of fibre).

Regarding the contribution of quinoa grains to nutrient intake in vegetarian and vegan diets, it must be underlined that these products can be the main source of protein. People following a vegan diet generally have an inadequate intake of some amino acids, such as phenylalanine, histidine, isoleucine, leucine, methionine, lysine, methionine, threonine, tryptophan, and valine. The consumption of quinoa allows obtaining a good balance of amino acids, as well as a proper intake of some B-group vitamins that may not be supplied in sufficient quantities by vegetarian or vegan diets [24,25].

#### 3.2.2. Flours

Quinoa flour, as a product of quinoa primary processing, is also available on the Italian market and represents 5% of the total amount of dataset food products (Figure 1). It was observed that both 100% quinoa flour and bread flour blends are available. Quinoa is generally included at 3% in blends with buckwheat, amaranth, and emmer.

The limited offering of quinoa flour products is likely related to the fact that quinoa flour incorporation in products, such as bakery ones, must be limited because of its high water adsorption properties and the subsequent scarce technological and organoleptic result. However, it is worth mentioning that pseudocereals are mostly milled to wholemeal flour [18], as their seeds are rather small. The addition of quinoa flour to bread/pasta formulations can hence represent an advantage in terms of nutritional quality: an increased consumption of wholemeal flour is, in fact, recommended by dietary guidelines worldwide.

At this regard, the analysis of nutrition facts showed a median DF content of 4.50 g/100 g, which is lower than the DF content generally reported in the literature for wholegrain flour (10.31 g/100 g) but higher than that for white flour (1.60 g/100 g) [26]. In Figure 3, it can be observed that the median and the first quartile of DF content in flours are higher than 3 g/100 g, which is the reference value for the nutritional claim “source of fibre”. Some items also have a content higher than 6 g/100 g, which is the reference value for the nutritional claim “high fibre”.

As regards macronutrients, it emerged that the median fat content (4.55 g/100 g) (Table 2) of quinoa flour is higher than the values available in the literature [26] for wholegrain flour and standard wheat flour (2.41 and 1.60 g/100 g, respectively). The median protein content (12.50 g/100 g) is comparable with values reported in the literature [26] for wholegrain flour (12.73 g/100 g) and higher than those regarding standard flour (11.54 g/100 g). Figure 2 shows that, in flours, the energy from protein is higher than the reference value for the claim “source of protein”. The inclusion of quinoa flour and/or blends thereof in homemade bread/pasta formulations may therefore contribute to increasing the protein content of the final product, while also providing essential amino acids.

The analysis of claims on the packaging showed that NC are only available in combination with SEC (13%) and with the GF, vegan, and/or organic logos (NC/AIC/SEC; 38%). “Source of fibre”, “High fibre”, and “Source of [vitamins] and/or [minerals]” are the displayed claims. The claim “source of protein” was never reported, despite it being observed in the nutrition declaration that the energy from protein in flours is always higher than 12% (Figure 2).

The GF logo was displayed in five out of eight products. Like NC, the GF logo is never present alone on the packaging but in combination with SEC and NC. This combination of claims may contribute to conveying to the consumer an idea that GF products are healthier than gluten-containing ones, though it has been found that GF products may have some nutrient inadequacies, such as high fat and salt contents [23].

Social and ethical logos occur in the packaging of seven out of eight flours (Table 1).

#### 3.2.3. Pasta Products and Couscous

Quinoa pasta products represent 23% of the total number of products included in our dataset (Figure 1). Both GF and gluten-containing (GC) pasta were available. In GF formulations, quinoa flour is added to blends of (whole) rice and maize at a range of 1 to 30%, whereas in GC pasta formulations, quinoa is added to durum wheat or whole grain emmer at a lower level, not higher than 20%. According to the scientific literature, the inclusion of pseudocereals, such as quinoa, in GF pasta formulations can have undesired organoleptic effects, such as a decrease in firmness and cooking loss [27]. The optimization of flour blends allows, nevertheless, minimizing the abovementioned negative effects [27]. The highly variability of quinoa addition is hence likely related to this technological aspect.

The analysis of nutritional declaration of quinoa pasta products (Table 2) showed that their composition is comparable with plain wheat pasta [28]. The median energy content in quinoa pasta is 355 kcal/100 g (Table 2), whereas plain wheat pasta provides 342 kcal/100 g [28]. As regards macronutrients, the median content of total fat, carbohydrates, and protein in quinoa pasta is comparable with the content reported in the literature for plain wheat pasta. The latter has 1.8 g/100 g total fat, 74 g/100 g total carbohydrates, and 10 g/100 g protein [28]. The median DF content in quinoa pasta was 2.40 g/100 g (Table 2), whereas 2.90 g/100 g was reported for plain wheat pasta [28].

The boxplots for energy from protein (Figure 2) and for dietary fibre (Figure 3) show that quinoa addition to pasta formulations does not entail an increase in protein and DF content. This may be due to the low percentage of quinoa addition and is despite the analysis of the quinoa flour category, which showed that these raw materials are a source of both protein and DF. The addition of quinoa to GF pasta formulations is advantageous to have a diversified diet but not to tackle the nutrient inadequacies of people adhering to a GF diet.

As regards claims on pasta packaging, it emerged that quinoa pasta is mostly claimed as “gluten-free”. A total of 26% of products displayed only AIC, 43% showed both AIC and SEC, and 11% presented AIC, NC, and SEC (Table 1).

As for nutrition claims, pasta products displayed them only in combination with the vegan and organic logos (Table 1).

#### 3.2.4. Bread and Bread Substitutes

Quinoa bread represents 8% of the total number of products included in our dataset, whereas bread substitutes represent 16% (Figure 1). Savoury snack crackers, puffed crackers, and extrudates were categorized as bread substitutes since they are sometimes consumed as bread alternatives during main meals. In recent decades, the consumption of bread in Italy has decreased more and more because it is perceived as fattening [29]. Bread substitutes, commonly available in small servings and/or with functional ingredients added, are thus preferred. In quinoa bread, quinoa is added to formulation at 1 to 8% of total ingredients, which is lower than the threshold level (25%) reported in literature to have an acceptable organoleptic quality of the final product [18]. In bread substitutes, quinoa addition levels showed a great variability. In savoury snack crackers, quinoa is generally added at 0.5 up to 13% to blends of amaranth and/or rice flour; in puffed crackers, quinoa addition ranges between 4 and 12.4% of blends of maize and (wholegrain) rice flours. In two products, quinoa is 70% of the total formulation.

Some bread substitutes are extrudates where quinoa is present at a low amount and is generally blended with maize and rice flours. Pseudocereals have high lipid content and food industry extruders produce well-expanded products when flours or blends thereof have a maximum fat content of about 5% [18]. Quinoa is therefore blended with rice and maize that have a lower fat content and thus require lower shear forces compared with quinoa flour [18].

As regards the nutrition declaration, median energy contents of 258 and 385 kcal/100 g were observed for bread and bread substitutes, respectively (Table 2). In the scientific literature, energy values lower than the first quartile and higher than the third quartile of quinoa bread and bread substitutes (Table 2) are reported. For plain wheat bread, which is generally considered a “medium” calorie food, an energy content of 220 kcal/100 g was found [30]. For packed bread and bread substitutes available on the Italian market, median energy contents of 274 and 1020 kcal/100 g, respectively, were reported [31].

As for macronutrients, it was observed that the two products categories significantly (*p* < 0.05) differ in median content of fat, carbohydrates, sugars, protein, and salt (Table 2). No significant differences (*p* < 0.05) were observed for saturates (Table 2).

The median fat content for quinoa-containing bread was 4.95 g/100 g (Table 2). This value is 10-fold higher than the content (0.4 g/100 g) reported for plain wheat bread in the Italian Food Composition database [22,32] but is in keeping with the values found by Angelino et al. for packed bread (4.4 g/100 g) [31]. The median fat content of quinoa-containing bread substitutes (3.15 g/100 g; Table 2) is lower than the one reported by Angelino et al. for bread substitutes (9.6 g/100 g) [31].

The median values for carbohydrates and sugars in quinoa-containing bread were 41 and 3.25 g/100 g, respectively. The carbohydrate content is in keeping with the content reported in the aforementioned study on the nutritional quality of bread products [31], whereas the median sugar content is lower in quinoa-containing bread (Table 2). It is possible to speculate that, following WHO recommendations, increasing efforts are being made by the food industry to reduce the intake of sugars [23]. In quinoa-containing bread substitutes, the median carbohydrate and sugar contents were 74.15 and 0.95 g/100 g, respectively (Table 2). Additionally, in bread substitutes, carbohydrate content was comparable with the values reported by Angelino et al., whereas sugar content was lower [31].

As regards protein and dietary fibre content, Figure 2 shows that the boxplot of quinoa-containing bread is completely below the reference value for the claim “source of protein”, whereas in bread-substitutes, only the median and the first quartile are below the reference value. As observed for quinoa-containing pasta, it is likely that quinoa flour is added at a percentage that is too low to significantly increase the bread’s protein content. On the other hand, Figure 3 shows that in quinoa-containing bread, the values of the median and first quartile of DF content are higher than the reference values for the NC “high fibre”, and in quinoa containing bread-substitutes the median content and the median and third quartile lies between the reference value for the NC “source of fibre” and the value for the “high fibre” claim.

The analysis of the claims reported on the packaging of quinoa-containing bread and bread substitutes showed that NC are never present alone on the packaging of these two product categories (Table 1). In bread, the NC “source of fibre” was available in only one product, in combination with the organic and GF logos (NC/AIC/SEC; Table 1). Based on the DF content distribution shown in Figure 3, it can be postulated that a higher number of bread products should be labelled as “high fibre”. In bread substitutes, NC are present on the packaging of 12 out of 24 products (Table 1) in combination with AIC and AIC/SEC. It emerged that NC other than “source of protein” and “source of fibre” are applied to bread substitutes, i.e., “low fat”, “low saturated fat”, “low sodium”, “high in phosphorus”, and “high in magnesium”.

As regards the presence of AIC on the packaging, 92% of quinoa-containing bread (11 out of 12 items) and 83% of quinoa-containing bread substitutes (20 out of 24 items) were claimed as GF (Table 1). In bread, the GF never appeared in combination with NC and SEC. As mentioned above, it could be nevertheless important for coeliac subjects, who generally report DF inadequacies, to find the claim “high fibre” on the packaging of GF quinoa-containing bread, considering that the nutrition declaration confirms this fact (Table 2; Figure 3).

The formulation of GF bread with quinoa and its availability on the market promotes a healthier diet for coeliacs. GF bread, formulated with corn and rice starch, generally has a high glycemic index (GI) [33], thus contributing to increasing the risk of developing metabolic syndromes, as shown by epidemiological studies [34]; the inclusion of quinoa can reduce bread’s GI value [34]. Hence, the formulation of GF bread with quinoa offsets the effects of GF cereal flours on the GI.

SEC are rarely present on the packaging of quinoa-containing bread (Table 1). Only 2 out of 12 bread products are claimed as organic. A higher percentage of SEC was found in quinoa-containing bread substitutes (16 out of 24 items; Table 1); the vegan and organic logos appear in combination with NC, AIC, and NC/AIC.

#### 3.2.5. Breakfast Cereals

Quinoa-based breakfast cereals represent 9% of the whole dataset, and puffed and flaked quinoa are the main sub-categories. In the different types of breakfast cereals, quinoa addition ranges between 7% and 100% in puffed quinoa.

The analysis of the nutrition declaration showed that the median energy content is 379 kcal/100 g (Table 2), which is line with the median content (385 kcal/100 g) reported by Angelino et al. for breakfast cereals available on the Italian market [35] and in keeping with other data available in the literature for sugared or fortified corn flakes [36]. No significant (*p* < 0.05) differences were observed for this parameter among the two main sub-categories (Table 2).

As regards macronutrients, the analysis of nutrition facts showed that quinoa-based breakfast cereals have a median content of total fat, total carbohydrates, and protein comparable with the content reported by Angelino et al. [35] but a higher content than wheat-based flakes consumed in the United States [36]. The median content for saturates and sugars is lower than that reported by Angelino et al. [35].

As regards macronutrient differences among the two subcategories, it was observed that flaked quinoa has a higher content of total carbohydrates than puffed quinoa (Table 2). No significant (*p* > 0.05) differences were observed between flaked and puffed quinoa for the median content of total fats, saturates, sugars, and protein (Table 2). Angelino et al. also report no significant differences (*p* > 0.05) between flakes and puffed cereals for all macronutrients [35]. Significant (*p* < 0.05) differences between the two categories were observed for salt content (Table 2).

The median DF content of quinoa-based breakfast cereals is 5.00 g/100 g (Table 2), which is higher than conventional fortified corn flakes consumed in the United States and lower than the DF content in fortified wheat and all bran cereals consumed in the United States [36]. Significant (*p* < 0.05) differences were observed between puffed and flaked quinoa. The median DF content was higher in puffed than flaked quinoa (Table 2).

As regards the distribution of quinoa-based breakfast cereals for DF, Figure 3 shows that the first quartile and the median of the boxplot are higher than the reference value for “source of fibre” and lower than the reference value for “high fibre”; the third quartile is higher than the reference value for the “high fibre” claim (Figure 3). As regards the distribution of quinoa-based breakfast cereals for protein, Figure 2 shows that the first quartile and the median content of protein are below the reference value for the NC “source of protein”.

As regards the presence of NC on the packaging of quinoa-based breakfast cereals, it was observed that they are present in 3 out of 14 products’ packaging in combination with SEC and AIC/SEC (Table 1). Quinoa breakfast cereals are labelled as a source of fibre and protein, but other claims, such as “low fat”, “with no added sugar” and “contains magnesium and phosphorus”, are also available. The scarce presence in the packaging of the “source of protein” claim is due to the low content for protein; in fact, quinoa puffing can reduce proteins and essential fatty acids [18]. 

AIC are displayed in the packaging of 4 out of 14 breakfast cereals, with the “organic” logo (AIC/SEC) and with NC and SEC (NC/AIC/SEC) (Table 1). SEC are the claims mostly available on the packaging of quinoa-based breakfast cereals. They are present alone in 7 out of 14 items and in combination with NC (1 item), AIC (2 items), and with NC/AIC (1 item) (Table 1).

#### 3.2.6. Ready-to-Cook Products

The demand for ready-to-eat and ready-to-cook foods is more and more on the rise because of the convenience of their utilization. Quinoa is also available as ready-to-cook products (Figure 1). The Italian market offers salads made up of cooked quinoa and other (pseudo)cereals and vegetables, as well as burgers of quinoa and vegetables. The availability of vegetable- and quinoa-containing salads follows up on consumer awareness of the need for consuming daily vegetables and grains such as legumes and pseudocereals for a healthy diet. Quinoa-containing burgers confirm the new trend for the market to satisfy the demand for alternative protein sources and the interest in plant proteins, as well as the enrichment of foods with sources of DF. In these products, quinoa is added at 10 to 20%.

Because of the heterogeneity of this food category, it is difficult to define a nutritional profile and make a comparison with the other categories of quinoa products. However, it can be observed that quinoa is generally included in low-energy ready-to-cook products, with a median energy content of 171 kcal/100 g (Table 2).

The packaging of quinoa ready-to-cook products mainly presents NC, either alone or in combination with SEC (Table 1). The most common are “source of fibre” and “source of protein”. Figure 2 shows that the median energy from protein is higher than the reference value for the “source of protein” claim. Figure 3 illustrates that the median DF content is higher than the “source of fibre” claim.

No AIC are displayed; this is likely due to the fact that processed products are hidden sources of gluten. This protein is in fact extensively used by the food industry as emulsifier, thickener, filler, fortifier, and flavour enhancer [37].

#### 3.2.7. Minor Categories: Snacks, Biscuits, and Yoghurt

Biscuits, snacks (e.g., single-serve multigrain bars), and yogurt represent only 2%, 3%, and 1% of the whole dataset, respectively. According to the list of ingredients, quinoa is present at 5 to 20% in biscuits; 2 to 22% in snacks, and 1 to 3% in yogurt.

Snacks and biscuits only show the “organic” logo in some cases, whereas one yogurt product is claimed as a source of fibre. The packaging of quinoa-containing snacks, biscuits, and yogurts never shows the GF logo. As for nutrition composition, the median content of macronutrients is generally high. However, the low number of items collected for these categories does not allow making important considerations about the nutritional composition.

### 3.3. Comparison between the Nutritional Composition of GF-Labelled and Gluten-Containing Quinoa Products

The gluten-free diet (GFD) entails the substitution of gluten-containing products with gluten-free products. Over recent decades the nutritional profile of GF food products has been increasingly investigated by the scientific community. Studies have highlighted that the nutritional profile of gluten-free-rendered products available on the market are low in protein content and high in fat and salt content in comparison with gluten-containing (GC) products [23]. Levels of dietary fibre and sugars in GF products are more adequate than in the past [23] but usually do not meet the dietary recommendations.

The comparison between the content of the above-mentioned components in GF-labelled quinoa products and quinoa products with no GF claim allowed obtaining an overview of the contribution of pseudocereals, such as quinoa, to the formulation of products.

Comparison between the two groups (GF vs. GC) was not possible for all food categories because in some of them (i.e., bread, biscuits, ready-to-cook products, snacks, and yogurts) only one GF or GC item or no GF items are available.

As regards the median protein content, Table 3 shows that no significant (*p* > 0.05) differences exist between GF and GC items in the categories of bread substitutes, breakfast cereals, and flours, whereas significant (*p* < 0.05) differences were observed for grains and pasta products. GF-labelled quinoa grains have a higher median protein content; this is due to the fact that 100% quinoa is mostly claimed as GF, whereas within the GC group there are mixtures of quinoa and cereals, such as rice or emmer, which are lower in protein content than pseudocereals. As regards quinoa-containing pasta, GF-labelled pasta showed a lower protein content (Table 3) and this is due to flour blends: GF-pasta is generally formulated with rice and maize flours, whereas plain pasta is formulated with durum wheat, which has a higher protein content than rice and maize.

No significant (*p* > 0.05) differences exist in the median fat content of GF and GC items included in the breakfast cereals, flours, and pasta categories (Table 3). On the contrary, the analysis of the nutrition facts for total fats in bread substitutes showed that GC items have a higher content than GF products (Table 3). This confirms the efforts of the GF food industry to reduce the fat content of GF food products. The median saturates content of GC bread substitutes was also higher than for GF products (Table 3). Our observations for fat content and saturates in GF vs. GC quinoa-containing bread substitutes were in keeping with the study by Angelino et al. on plain bread substitutes [31]. In grains, GF items had a higher content in total fat than the mixtures with GC grains. As mentioned above, total lipid content is generally higher in pseudocereals, such as quinoa (4.0–7.6%) [18], than in grains, such as wheat. However, it is worth highlighting that unsaturated fatty acids (e.g., linoleic and oleic acids) are the highest proportion of lipids (71.0–84.5% in quinoa) [18]. The median saturates content was, in fact, not significantly different between GF and GC items (Table 3).

Median sugar content was significantly (*p* < 0.05) lower in GF quinoa-containing bread substitutes and pasta than in GC ones, whereas no significant differences were observed between GC and GF items in breakfast cereals, flours, and grains (Table 3). The data thus confirm an opposite trend for GF products than in the past. Our findings for quinoa bread substitutes were in keeping with the aforementioned study by Angelino et al. on bread substitutes. As a matter of fact, they also found that the median content of sugars was lower in GF than in GC bread substitutes [31].

The median content of dietary fibre was comparable for GC and GF products in bread substitutes, breakfast cereals, flours, and grains. In pasta, GC products were richer in DF than GF pasta products. Major efforts are therefore necessary to increase the DF content in GF pasta.

No significant differences were found in the median salt content of GF versus GC products in breakfast cereals, flours, grains, and pasta (Table 3). GF bread substitutes were, on the other hand, a more minor source of salt than GC items. Angelino et al. also observed a lower median salt content in GF-labelled bread substitutes surveyed in their study than in non-GF products [31].

## 4. Conclusions

Food products formulated with quinoa are present in the Italian market in a wide range of food categories, varying from plain grains to ready-to-cook products. It was observed that in some of them quinoa is the primary ingredient (>50%), in accordance with the rules laid down by Regulation (EU) No. 1169/2011 [13], whereas in other products, including pasta and bread, it is added at a very low content. It might be useful for the consumer to define a minimum level for the inclusion of the word “quinoa” in the descriptive name.

Claims on the packaging provide information on the quality of quinoa food products to the consumer. Nutrition claims are commonly present in combination with allergy and intolerance claims or with social and ethical claims. Based on the nutrition facts, a higher proportion of products are eligible for nutrition claims.

Some quinoa products showed an undesirable content for some nutrients, such as the fat content in bread. The analysis of the nutritional composition of quinoa GF products versus non-GF-labelled products showed that the nutritional profile of quinoa GF-labelled products is more compliant to the worldwide nutritional recommendations than in the past.

The study included a moderate number of products, which were in some food categories not suitable for elaborating any opinion on the nutritional quality of the category itself. This is due to a low availability of food products formulated with novel ingredients, such as quinoa. It might be interesting to update this survey in the near future to investigate if a higher number of quinoa food products are available and if these findings are confirmed.

## Figures and Tables

**Table 1:** Distribution of claims across the main product categories.

Product Category	Items Per Category		Products with						
		No Claims	NC	AIC	SEC	NC/AIC	NC/SEC	AIC/SEC	NC/AIC/SEC
	*No. (%)* *	*No. (%)* **	*No. (%)* **	*No. (%)* **	*No. (%)* **	*No. (%)* **	*No. (%)* **	*No. (%)* **	*No. (%)* **
Grains	42 (28%)	8 (19%)	2 (5%)	0 (0%)	11 (26%)	1 (2%)	3 (7%)	10 (24%)	7 (17%)
Flours	8 (5%)	1 (13%)	0 (0%)	0 (0%)	1 (13%)	0 (0%)	1 (13%)	2 (25%)	3 (38%)
Pasta/couscous	35 (23%)	0 (0%)	0 (0%)	9 (26%)	6 (17%)	0 (0%)	1 (3%)	15 (43%)	4 (11%)
Bread	12 (8%)	0 (0%)	0 (0%)	10 (84%)	1 (8%)	0 (0%)	0 (0%)	0 (0%)	1 (8%)
Bread substitutes	24 (16%)	3 (12%)	0 (0%)	2 (8%)	0 (0%)	3 (13%)	1 (4%)	6 (25%)	9 (38%)
Breakfast cereals	14 (9%)	1 (7%)	0 (0%)	1 (7%)	7 (50%)	0 (0%)	2 (14%)	2 (14%)	1 (7%)
Ready-to-cook products	8 (5%)	3 (38%)	3 (38%)	0 (0%)	1 (13%)	0 (0%)	1 (13%)	0 (0%)	0 (0%)
Snacks	4 (3%)	2 (50%)	0 (0%)	0 (0%)	1 (25%)	0 (0%)	0 (0%)	1 (25%)	0 (0%)
Biscuits	3 (2%)	1 (33%)	0 (0%)	0 (0%)	2 (67%)	0 (0%)	0 (0%)	0 (0%)	0 (0%)
Yogurt	2 (1%)	1 (50%)	1 (50%)	0 (0%)	0 (0%)	0 (0%)	0 (0%)	0 (0%)	0 (0%)
TOTAL		19 (13%)	6 (4%)	22 (15%)	30 (20%)	4 (3%)	8 (5%)	36 (24%)	25 (17%)

NC: nutrition claim; AIC: allergy and/or intolerance claim; SEC: social and/or ethical claim; NC/AIC: nutrition plus allergy and intolerance claims; NC/SEC: nutrition plus social and ethical claims; AIC/SEC: allergy and intolerance plus social and ethical claims; NC/AIC/SEC: nutrition, allergy and intolerance and social and ethical claims. (%) *: % versus total; (%) **: % within the product category.

**Table 2:** Mandatory nutrition declaration across product categories.

Product Category	Subcategory	Energy	Total Fat	Saturates	Total Carbohydrates	Sugars	Protein	Dietary Fibre	Salt
		kJ/100 g kcal/100 g	g/100 g	g/100 g	g/100 g	g/100 g	g/100 g	g/100 g	g/100 g
Grains	Total	1526 (1458–1574) 362 (344–375)	5.80 (3.02–6.10)	0.60 (0.50–0.70)	62.60 (59.55–65)	2.40 (0.50–3.50)	12.20 (9.70–13.83)	6.95 (4.38–8.70)	0.01 (0.01–0.04)
	*Dry quinoa*	1550 (1490–1594) 368 (355–380) ^a^	5.90 (5.40–6.20) ^a^	0.70 (0.52–0.70) ^a^	62.40 (60.10–64.20) ^a^	3.10 (1.60–3.75) ^a,A^	12.40 (12.05–14.05) ^a^	7.00 (6.70–8.85) ^a^	0.01 (0.00–0.40) ^a^
	*Mixtures*	1461 (786–1488) 345 (186–351) ^b,A^	2.70 (1.65–3.95) ^b,A^	0.50 (0.30–0.60) ^b,A^	66.60 (36.35–71.85) ^a,A^	0.90 (0.40–1.75) ^b^	8.00 (4.95–10.15) ^b,A^	4.50 (2.25–6.40) ^b,A^	0.00 (0.00–0.40) ^a,A^
	*Canned*/*steamed quinoa*	311 (178–1232) 79 (42–295) ^B^	1.20 (0.15–4.13) ^A^	0.10 (0.00–0.50) ^A^	37.40 (9.57–68.97) ^A^	2.15 (0.13–9.72) ^A^	3.35 (1.75–12.22) ^A^	2.65 (2.42–10.30) ^A^	0.75 (0.25–1.250) ^A^
Flours		1513 (1500–1591) 359 (354–375)	4.55 (1.97–6.37)	0.55 (0.30–0.95)	71.41 (57.72–75.45)	1.90 (1.70–3.75)	12.50 (11.92–13.20)	4.50 (4.40–9.65)	0.01 (0.01–0.15)
Pasta/ couscous		1499 (1483–1542) 355 (352–364)	2.00 (1.40–2.70)	0.40 (0.30–0.60)	74.60 (70.00–77.80)	2.40 (0.50–3.50)	8.4 (7.30–11.00)	2.40 (1.00–3.30)	0.01 (0.00–0.03)
Bread		1084 (980.30–1116.30) 258 (233.00–265.75) ^b^	4.95 (3.48–7.40) ^a^	0.70 (0.50–0.95) ^a^	41.00 (36.50–44.50) ^b^	3.25 (2.43–4.37) ^a^	4.25 (3.35–5.02) ^b^	7.55 (6.27–8.77) ^a^	1.05 (1.00–1.20) ^a^
Bread substitutes		1632 (1543.80–1735.40) 385 (368–414) ^a^	3.15 (2.25–8.10) ^b^	0.60 (0.30–1.20) ^a^	74.15 (68.10–77.91) ^a^	0.95 (0.57–1.30) ^b^	9.40 (8.10–11.40) ^a^	3.80 (2.84–4.52) ^b^	0.47 (0.29–1.27) ^b^
Breakfast cereals	Total	1603.5 (1547–1669) 379 (371–400)	4.50 (2.60–5.45)	0.40 (0.20–0.62)	72.00 (64.30–78.25)	4.20 (1.60–7.78)	10.85 (9.00–13.25)	5.00 (3.80–7.45)	0.05 (0.00–1.00)
	*Flaked*	1605 (1597–1664) 386 (379–393) ^a^	2.80 (2.60–3.00) ^a^	0.30 (0.20–0.40) ^a^	79.00 (77.00–81.00) ^a^	6.85 (4.87–7.70) ^a^	9.35 (9.00–9.70) ^a^	3.60 (3.20–4.00) ^b^	1.70 (1.00–2.40) ^a^
	*Puffed*	1517 (1435–1571) 376 (359–407) ^a^	4.90 (3.50–6.80) ^a^	0.50 (0.30–0.85) ^a^	66.00 (63.20–72.00) ^b^	3.50 (1.30–5.95) ^a^	12.80 (10.50–14.30) ^a^	5.50 (5.00–11.10) ^a^	0.01 (0.00–0.12) ^b^
Ready-to-cook products		717 (638–1171) 171 (152–277)	3.85 (2.25–7.32)	0.55 (0.32–1.07)	24.30 (13.82–51.15)	1.60 (0.39–3.80)	5.80 (4.60–10.75)	3.60 (2.60–6.18)	0.79 (0.14–1.4)
Biscuits		1986 (1812–2044) 468 (431–473)	19.00 (14.00–20.00)	3.20 (2.10–4.10)	68.00 (67.00–69.00)	24.00 (20.00–25.00)	6.70 (6.10–7.20)	2.70 (2.50–3.00)	0.50 (0.28–0.68)
Snacks		1906 417	16.75 (12.63–28.38)	5.90 (1.77–7.60)	62.00 (36.98–64.75)	12.25 (1.63–31.50)	6.75 (5.47–9.22)	5.50 (4.35–9.65)	0.48 (0.09–1.47)
Yogurt		449 146	3.70	1.75	20.35	15.25	6.70	2.30	0.19

Values are median (25th–75th percentile). Different lowercase or uppercase letters between subcategories of grains and breakfast cereals and between bread and bread substitutes indicate significant (*p* < 0.05) differences (Kruskal–Wallis non-parametric one-way ANOVA for independent samples with multiple pairwise comparisons; Mann–Whitney non-parametric test for two independent samples).

**Table 3:** Protein, fat, sugars, dietary fibre and salt content across the five food categories with both gluten-free and gluten-containing items.

Product Category		No. of Items	Protein	Fat	Saturates	Sugars	Dietary Fibre	Salt
			*g/100 g*	*g/100 g*	*g/100 g*	*g/100 g*	*g/100 g*	*g/100 g*
Bread substitutes	GF	19	9.30 (8.10–11.10) ^a^	3.03 (2.10–4.00) ^b^	0.60 (0.30–0.80) ^b^	0.70 (0.50–1.30) ^b^	3.50 (2.84–4.00) ^a^	0.40 (00.26–1.10) ^b^
	GC	03	11.90 (11.00–13.00) ^a^	12.60 (11.00–13.00) ^a^	1.70 (1.40–1.90) ^a^	3.20 (1.30–4.00) ^a^	4.60 (3.90–6.70) ^a^	1.70 (1.50–1.90) ^a^
Breakfast cereals	GF	04	11.10 (9.70–12.88) ^a^	3.55 (2.60–4.50) ^a^	0.30 (0.20–0.40) ^a^	4.65 (1.60–7.70) ^a^	4.10 (3.20–5.00) ^a^	1.21 (0.00–2.40) ^a^
	GC	10	10.50 (8.50–14.15) ^a^	4.95 (2.87–6.65) ^a^	0.50 (0.35–0.77) ^a^	4.20 (2.88–8.75) ^a^	5.25 (4.00–9.80) ^a^	0.06 (0.00–0.81) ^a^
Flours	GF	05	13.00 (11.25–13.20) ^a^	6.00 (3.10–6.60) ^a^	0.80 (0.30–1.30) ^a^	3.60 (1.70–5.05) ^a^	4.50 (4.25–9.00) ^a^	0.01 (0.01–0.56) ^a^
	GC	03	12.00 (12.00–15.00) ^a^	1.60 (1.60–6.50) ^a^	0.30 (0.30–1.00) ^a^	1.90 (0.50–1.90) ^a^	4.40 (4.40–15.00) ^a^	0.00 (0.00–0.19) ^a^
Grains	GF	18	12.95 (12.20–14.10) ^a^	5.90 (5.00–6.25) ^a^	0.65 (0.50–0.70) ^a^	2.20 (0.10–3.40) ^a^	6.95 (6.60–7.43) ^a^	0.01 (0.01–0.02) ^a^
	GC	24	11.35 (6.18–13.45) ^b^	4.90 (2.12–6.00) ^b^	0.60 (0.35–0.70) ^a^	2.80 (0.60–3.88) ^a^	6.90 (3.20–9.00) ^a^	0.01 (0.01–0.28) ^a^
Pasta	GF	29	8.16 (7.30–9.00) ^b^	1.50 (1.20–2.80) ^a^	0.50 (0.30–0.72) ^a^	1.00 (0.39–3.80) ^b^	2.10 (1.00–3.30) ^b^	0.01 (0.00–0.08) ^a^
	GC	06	12.00 (11.83–12.25) ^a^	2.20 (2.12–2.32) ^a^	0.00 (0.00–0.42) ^b^	3.00 (3.00–3.88) ^a^	3.00 (3.00–5.13) ^a^	0.00 (0.00–0.01) ^a^

Values are median (25th–75th percentile). For each product category, different lowercase letters in the pair GC–GF of the same component indicate significant differences (Mann–Whitney non-parametric test for two independent samples), *p* < 0.05.

## Data Availability

The data presented in this study are available on request from the corresponding author.
